# Neglected tropical diseases: A DFID perspective

**DOI:** 10.1371/journal.pntd.0005492

**Published:** 2017-04-20

**Authors:** Charlotte Watts

**Affiliations:** Social and Mathematical Epidemiology, London School of Hygiene and Tropical Medicine, London, United Kingdom; University of Washington, UNITED STATES

NTDs affect over 1 billion people, and cause 170,000 deaths each year. They result in disability, stigma and disfigurement. NTDs also push families into poverty—the direct financial costs of illness due to visceral leishmaniasis exceed 20% of total annual household expenditure for 30% of households in Ethiopia, around a quarter of households in Bangladesh and Sudan, and 14% of households in India. These figures are stark, but there is important progress. In the past five years, the number of people at risk for NTDs fell by 20 percent–from 2 billion to 1.6 billion. Some countries are now able to stop mass drug administration for some NTDs. These are amazing achievements. Particularly in this, the 5^th^ anniversary of the London Declaration on NTDs, we need to celebrate these successes, and also collectively step up, to tackle remaining and new challenges.

Motivated by the need to tackle the impacts that NTDs have on the poor in the developing world, the UK has had a long history of NTD support, using its funding and influence to champion and help catalyse the academic, public and private sector to take action. As NTDs primarily occur in rural and poor urban areas of low-income and middle-income countries [1], NTDs not only provides an important marker for global progress towards achieving universal access to health, but also towards the leave no-one behind agenda, and the commitment that development should seek to improve the lives of the poorest and most vulnerable in the world.

Strong partnership has been central to the achievements to date. Effective partnership with endemic countries has been critical. Partnership with other donors, pharmaceutical companies, multilateral organisations such as WHO and the World Bank, and a wide range of organisations that provide support and technical assistance to NTD programmes on the ground, have also been central to successes to date. The UK and the USA have been the largest bilateral donors on NTD implementation, with the Bill and Melinda Gates Foundation also providing substantial support. The UK made a commitment to spend an additional £195m at the London Declaration on NTDs. Working in partnership with national governments, the UK supports programmes to tackle a range of NTDs. These include delivering over 136m treatments for the diseases tackled through mass drug administration in 2016; reducing disability by supporting surgery for hydrocoeles due to lymphatic filariasis and surgery to prevent blindness from trachoma. Funding has helped support Asia achieve elimination of visceral leishmaniasis as a public health problem; and supported African countries with visceral leishmaniasis to achieve better control, including the earlier detection and response of outbreaks. Support to the Carter Center has contributed to the eradication of Guinea worm disease: in 2016 there were 25 cases of GWD in only 3 countries down from over 3m a year in more than 20 countries when the programme started in 1986.

Our ability to tackle NTDs has been greatly enhanced by the availability of highly cost effective treatment options. Most people requiring a package of essential NTD medicines can be reached by mass treatment for less than US$0.50 per person. This includes treatment for schistosomiasis, trachoma, lymphatic filariasis, onchocerciasis and soil transmitted helminths. Most drugs have been donated by the pharmaceutical industry. Without this generous support, announced at the London Declaration, and valued at US$17.8 million to 2020, we would not have achieved the progress seen to date.

Looking forward, we need to look for opportunities to achieve synergies in programming. The seven preventive chemotherapy diseases (ascariasis, hookworm infections, lymphatic filariasis, onchocerciasis, schistosomiasis, trichuriasis, and trachoma) are preventable by a simple oral drug treatment, administered once or twice a year. Due to the geographic overlap of endemic areas for some NTDs, and a common requirement for preventive chemotherapy, an integrated approach to mass drug administration has been increasingly adopted. This integrated approach provides a means to make cost savings and increase efficiency compared to single disease approaches. We need to learn from and accelerate this approach, and identify what adaptations may be required as progress continues to be made. There are other areas also where integrated action can lead to multiple impacts. Improving access to clean water, hygiene and sanitation will help prevent trachoma and schistosomiasis, but will also have wider health benefits. The WHO Water, Sanitation and Hygiene strategy for accelerating and sustaining progress on NTDs is important, but we still need to learn the best ways to implement this.

Rigorous research and new innovation remains an important part of the NTD response. DFID supported NTD research includes both product development and operational research. This has resulted in new drugs for sleeping sickness, visceral leishmaniasis, Chagas disease and diagnostics for sleeping sickness through the work of Drugs for Neglected Diseases Initiative (DNDi) and Foundation for Innovative New Diagnostics respectively. DFID is also investing in development of new insecticides through Innovative Vector Control Consortium and implementation research on the integrated approach to mass drug administration for tackling NTDs through COUNTDOWN.

Progress will only continue to be made by being innovative, testing new technologies and approaches to programming, and assessing how established approaches may need to be adapted to respond to different epidemiological and geographic contexts. It is critical that new research findings–both positive and negative–are disseminated promptly, so that lessons can be learnt about what forms of intervention are effective, and those that are not.

Strong surveillance and monitoring will continue to form an important backbone of NTD programmes. As we make progress we need to modify and test new approaches to mapping and surveillance in low prevalence settings. New digital technologies may provide opportunities to do things differently, more rapidly, and more efficiently moving forward. We also need rigorous data on the impact of programmes on disease transmission and health burden, to help make the case for further investment. Though knowing we have delivered millions of treatments is important; information on reductions in prevalence or intensity of infections is vital. Similarly, we need to know whether countries are meeting the criteria to stop Mass drug administration either nationally or in some geographic areas. Rigorous cost effectiveness data will continue also to be important, to ensure that available resources are used for the greatest possible good.

With the successes to date, the NTD field will need to respond to the new epidemiological and contextual realities. The world’s populations are becoming increasingly urbanised, yet many established models of intervention have been tested and used in rural populations. For example, public school systems often provide an important platform for preventive therapy for schistosomiasis. We need to ensure that children in informal or private schools, and children who are not attending school, are getting the same access to preventive chemotherapy. We need to identify also how best to deliver effective interventions in challenging and complex settings–such as within fragile and conflict affected states, and to migrant and displaced populations. It is in these settings where many of the most vulnerable live, and where the burden of NTDs will increasingly be concentrated.

There has been much progress since the London Declaration. Yet NTD transmission continues in many countries, and hundreds of millions of people still do not have access to the treatments that they need. We cannot be complacent. If we are to achieve global targets we must continue to work in strong partnership, and rise to tackle new challenges. The research community will continue to have an essential role to play–generating and testing the new tools and rigorous evidence needed to ensure that the burden of NTDs can continue to be reduced, and that no-one is left behind.
